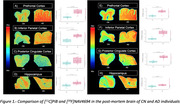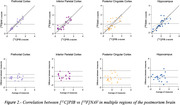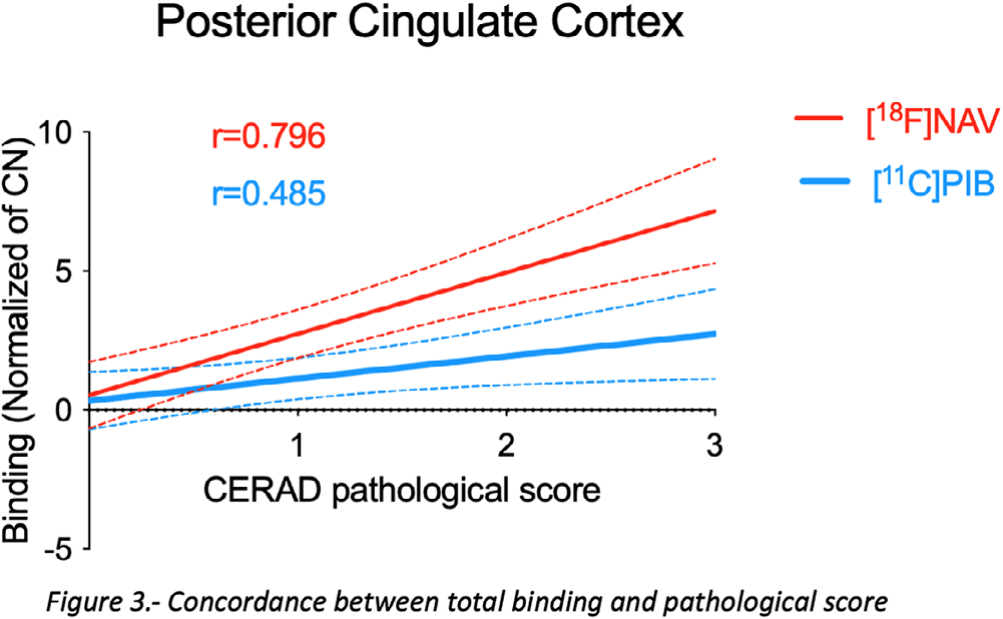# Autoradiographic comparison of [11C]PiB and [18F]NAV4694 in post‐mortem human brain tissue

**DOI:** 10.1002/alz.093262

**Published:** 2025-01-09

**Authors:** Antonio Aliaga, Joseph Therriault, Arturo Aliaga Aliaga, Kely Monica Quispialaya Socualaya, Jean‐Paul Soucy, Arthur C. Macedo, Giovanna Carello‐Collar, Marco De Bastiani, Nesrine Rahmouni, Tharick A. Pascoal, Eduardo R. Zimmer, Pedro Rosa‐Neto

**Affiliations:** ^1^ McGill University, Montreal, QC Canada; ^2^ Universidade Federal do Rio Grande do Sul, Porto Alegre, Rio Grande do Sul Brazil; ^3^ Translational Neuroimaging Laboratory, The McGill University Research Centre for Studies in Aging, Montréal, QC Canada; ^4^ McConnell Brain Imaging Centre ‐ McGill University, Montreal, QC Canada; ^5^ Montreal Neurological Institute, McGill University, Montreal, QC Canada; ^6^ Universidade Federal do Rio Grande do Sul, Porto Alegre, RS Brazil; ^7^ Departments of Psychiatry and Neurology, University of Pittsburgh School of Medicine, Pittsburgh, PA USA; ^8^ Federal University of Rio Grande do Sul (UFRGS), Porto Alegre, RS Brazil; ^9^ Brain Institute of Rio Grande Do Sul, PUCRS, Porto Alegre, RS Brazil; ^10^ Montreal Neurological Institute, Montréal, QC Canada

## Abstract

**Background:**

To evaluate the in vitro binding properties of [^11^C]PiB and [^18^F]NAV4694 head‐to‐head in post‐mortem human brain tissue.

**Method:**

Autoradiography was used to assess uptake of [^11^C]PiB and [^18^F]NAV4694 in control (CN) and Alzheimer’s disease (AD) autopsy‐confirmed brain tissues. The study focuses on the analysis of the prefrontal cortex, inferior parietal cortex, posterior cingulate cortex and hippocampus sections in 11 CN and 11 AD cases. The binding values of [^11^C]PIB and [^18^F]NAV4694 were calculated from regions of interest (ROIs) drawn manually in the mentioned sections. In addition, a histopathology study (CERAD) was performed in the posterior cingulate cortex section of 10 CN and 10 AD cases.

**Result:**

For both radiotracers investigated, we observed significant differences between CN and AD tissues uptakes in the prefrontal cortex, inferior parietal cortex, posterior cingulate cortex and hippocampus. Higher binding was detected for [^18^]NAV4694 compared to [^11^C]PIB in AD prefrontal, inferior parietal, and posterior cingulate cortices, while binding in the hippocampus was comparable for both radioligands. A strong correlation between [^18^]NAV4694 and [^11^C]PIB was found in the prefrontal cortex (R=0.934, p<0.0001) and inferior parietal cortex (R=0.833, p<0.0001), and lower values were obtained in the posterior cingulate cortex (R=0.699, p=0.0003) and hippocampus (R=0.72, p=0.0002). The analysis of correlation between the binding of [^11^C]PIB and [^18^F]NAV4694 in the posterior cingulate cortex section and the CERAD histopathology scores revealed a higher concordance for [^18^F]NAV4694 (R=0.796) compared to [^11^C]PIB (R=0.485).

**Conclusion:**

This study reports the first head‐to‐head comparison of [^11^C]PIB and [^18^F]NAV4694 in postmortem human brain tissue. [^11^C]PIB and [^18^F]NAV4694 showed high binding to amyloid beta aggregates in post‐mortem human brain tissues by autoradiography. Our results suggest that NAV4694 may have a higher effect size when differentiating between individuals with and without AD.